# Quantitative evaluation of meniscus injury using synthetic magnetic resonance imaging

**DOI:** 10.1186/s12891-024-07375-4

**Published:** 2024-04-15

**Authors:** Lingtao Zhang, Wenfeng Mai, Xukai Mo, Ruifen Zhang, Dong Zhang, Xing Zhong, Shuangquan Zhao, Changzheng Shi

**Affiliations:** 1 Medical Imaging Center, The First Affiliated Hospital of Jinan University, No. 613 West Huangpu Avenue, Tianhe District, Guangzhou, 510630 China; 2 UItrasonic Department, The First Affiliated Hospital of Jinan University, Guangzhou, China; 3 Medical Imaging Center, The Second Affiliated Hospital of Shenzhen University, No. 118 Longjing 2nd Road, Bao‘an District, Shenzhen, 518101 China; 4 Subingtian center for speed research and training, Guangdong Key Laboratory of speed capability research, School of physical education, Jinan University, Shenzhen, China

**Keywords:** Knee, Meniscus injury, MRI, SyMRI

## Abstract

**Background:**

Magnetic resonance imaging (MRI) can diagnose meniscal lesions anatomically, while quantitative MRI can reflect the changes of meniscal histology and biochemical structure. Our study aims to explore the association between the measurement values obtained from synthetic magnetic resonance imaging (SyMRI) and Stoller grades. Additionally, we aim to assess the diagnostic accuracy of SyMRI in determining the extent of meniscus injury. This potential accuracy could contribute to minimizing unnecessary invasive examinations and providing guidance for clinical treatment.

**Methods:**

Total of 60 (*n*=60) patients requiring knee arthroscopic surgery and 20 (*n*=20) healthy subjects were collected from July 2022 to November 2022. All subjects underwent conventional MRI and SyMRI. Manual measurements of the T1, T2 and proton density (PD) values were conducted for both normal menisci and the most severely affected position of injured menisci. These measurements corresponded to the Stoller grade of meniscus injuries observed in the conventional MRI. All patients and healthy subjects were divided into normal group, degeneration group and torn group according to the Stoller grade on conventional MRI. One-way analysis of variance (ANOVA) was employed to compare the T1, T2 and PD values of the meniscus among 3 groups. The accuracy of SyMRI in diagnosing meniscus injury was assessed by comparing the findings with arthroscopic observations. The diagnostic efficiency of meniscus degeneration and tear between conventional MRI and SyMRI were analyzed using McNemar test. Furthermore, a receiver operating characteristic curve (ROC curve) was constructed and the area under the curve (AUC) was utilized for evaluation.

**Results:**

According to the measurements of SyMRI, there was no statistical difference of T1 value or PD value measured by SyMRI among the normal group, degeneration group and torn group, while the difference of T2 value was statistically significant among 3 groups (*P*=0.001). The arthroscopic findings showed that 11 patients were meniscal degeneration and 49 patients were meniscal tears. The arthroscopic findings were used as the gold standard, and the difference of T1 and PD values among the 3 groups was not statistically significant, while the difference of T2 values (32.81±2.51 of normal group, 44.85±3.98 of degeneration group and 54.42±3.82 of torn group) was statistically significant (*P*=0.001). When the threshold of T2 value was 51.67 (ms), the maximum Yoden index was 0.787 and the AUC value was 0.934.

**Conclusions:**

The measurement values derived from SyMRI could reflect the Stoller grade, illustrating that SyMRI has good consistency with conventional MRI. Moreover, the notable consistency observed between SyMRI and arthroscopy suggests a potential role for SyMRI in guiding clinical diagnoses.

## Background

Meniscus injury is a common cause of knee pain, with an annual incidence of 60–70 per 100,000 [1]. The common causes of meniscal tears are trauma and degeneration [2]. The primary role of the meniscus is to distribute the load within the knee joint by increasing consistency, thereby reducing the stress on the articular cartilage [3]. Additionally, the meniscus contributes to secondary functions such as shock absorption, stability, lubrication and proprioception of the knee joint [4–6]. Magnetic resonance imaging (MRI) stands out as a valuable tool for meniscus injury, exhibiting an accuracy range from 82 to 95% [7]. Proton density-weighted image (PDWI) serves as the optimal sequence for evaluating meniscus tears [8]. At present, the Stoller grade is used as the MRI diagnostic standard for meniscus injury [9]. Other MRI techniques are also employed for diagnosing meniscus injuries. Williams et al. [10] used ultrashort echo time (UTE) -T2* mapping to study subclinical meniscus injury after anterior cruciate ligament (ACL) tear. Similarly, the T1 value of the meniscus calculated by delayed gadolinium-enhanced MRI of cartilage (dGEMRIC) has been shown to correlate with that of articular cartilage [11].

Synthetic magnetic resonance imaging (SyMRI) requires only a single scan of multiple dynamic multiple echo (MDME) to reconstruct images with many different contrasts [12]. By setting 2 echo times (TE) and 4 delay times (TD), echoes after different TE are collected. And T1 value, T2 value and PD value of tissue is calculated by the program, in order to obtain the quantitative image of the tissue. Various contrast images are reconstructed by setting different repetition time (TR), TE, inversion time, flip angle and other parameters with programs and software, such as T1-weighted image (T1WI), T2-weighted image (T2WI), short-tau inversion recovery (STIR), fluid attenuation inversion recovery (FLAIR), etc [13]. . . Compared with the qualitative diagnosis of conventional MRI, SyMRI is more objective and accurate in the evaluation of the disease. Several investigations that have used SyMRI in brain images have revealed acceptable image quality and artifacts, and suggested the clinical utility of the method [14, 15].

Currently, SyMRI is applied in various disease studies, including multiple sclerosis diagnosis [16], breast cancer invasion degree and prognosis evaluation [17], bone metastasis in prostate cancer [18], and the analysis of carotid atherosclerotic plaque composition [19]. Besides, SyMRI is also used in bone and joint research. Boudabbous et al. [20] found that SyMRI had good image quality as conventional MRI in the knee joint, but their study was a pilot feasibility study without quantitative analysis. Kumar et al. [21] found that similar detection rates happened in knee joint derangements for SyMRI and conventional imaging sequences. Additionally, the accuracy of synthetic double-inversion recovery imaging for detecting synovitis compared to that of T1-weighted CE-MRI was relatively good [22]. Besides, SyMRI was not in-depth and comprehensive enough in the study of knee joint lesions.

The aim of the study was to analyze the consistency of SyMRI and conventional MRI in diagnosing meniscal lesions. With arthroscopic findings during surgery as the gold standard, the study explored whether SyMRI could analyze the meniscal lesion quantificationally. Additionally, the difference in diagnostic efficiency between conventional MRI and SyMRI for meniscus degeneration and tear was discussed, with a view to reducing unnecessary arthroscopy and guiding clinical treatment.

## Materials and methods

### Subjects

**Fig. 1:**
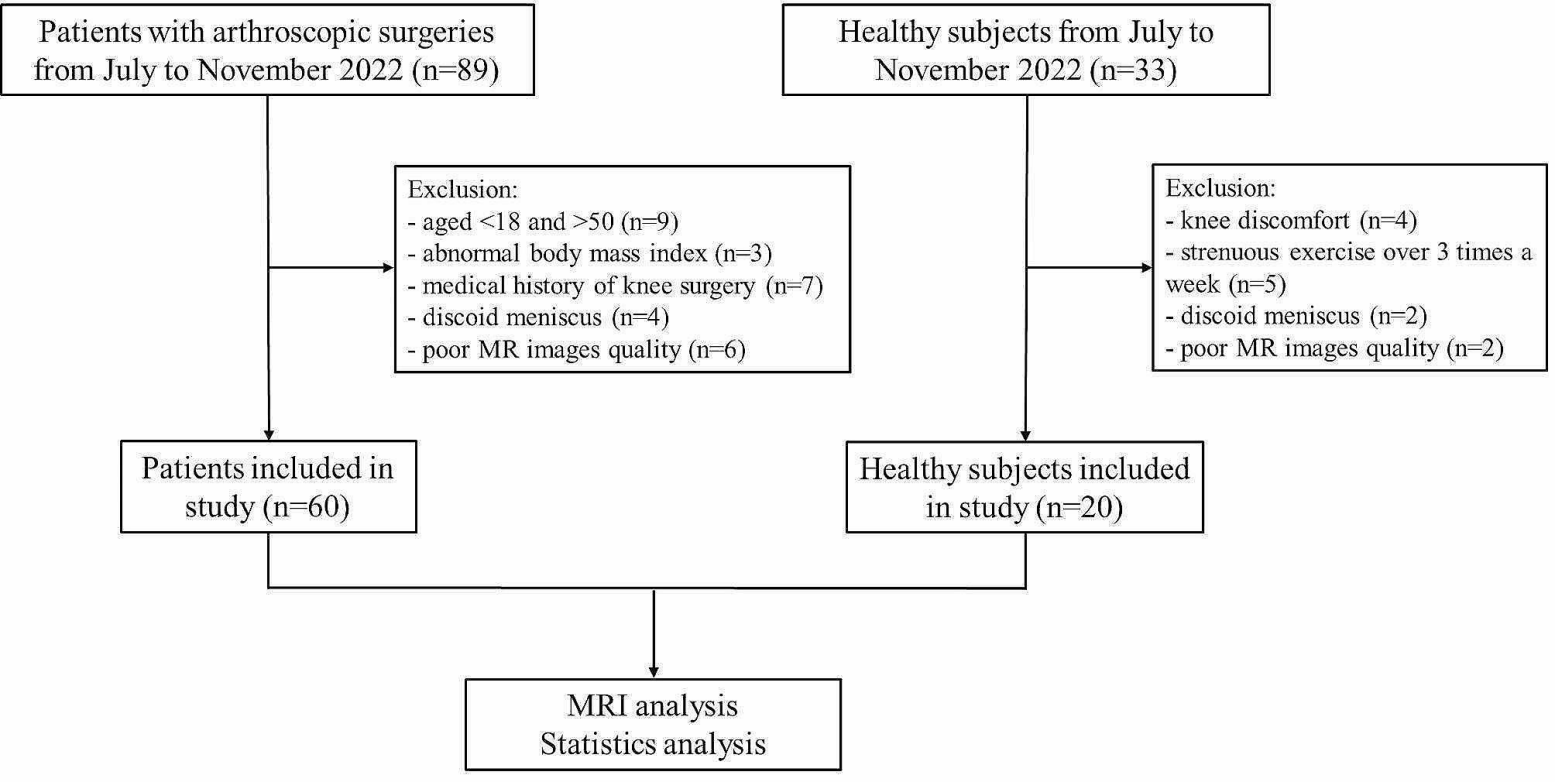
The selection process of the included patients and healthy subjects

The patients requiring arthroscopic surgery in the Ward of Bone Joint and Sports Medicine of the First Affiliated Hospital of Jinan University from July to November 2022 were collected continuously. All arthroscopic surgeries were performed by an experienced orthopedic surgeon with more than 15 years of experience working in department of orthopedics. The inclusion criteria encompassed individuals aged 18–50 years old with a recent history of knee pain or discomfort, as well as normal body mass index. Positive indications of knee discomfort included positive McMurray’s test, Lachman test, and inverse Lachman test. The exclusion criteria comprised individuals with a medical history of knee surgery, congenital dysplasia of the meniscus, inability to cooperate during the magnetic resonance (MR) scan, and medical conditions such as rheumatoid arthritis, gout, and ankylosing spondylitis. Besides, healthy subjects were collected. They were 18–30 years old with normal body mass index and the number of men and women included in the studies was similar. Healthy subjects had no symptoms of knee discomfort, no strenuous daily exercise, no underlying diseases and other factors affecting meniscus lesions, and conventional MRI showed no clear signs of meniscus injury. The selection process of the included patients and healthy subjects was displayed in Fig. 1.

### MRI

MR scans were conducted by the 3.0T MR scanner (Signa Premier, GE Healthcare, Waukesha, WI) and 16-channel surface flexible coil. Each subject was placed in a supine position with the feet in front and the lower limbs naturally extended. The positioning line was placed on the lower edge of the patella. The scan was performed by a radiologist skilled in MR operations.

**Fig. 2:**
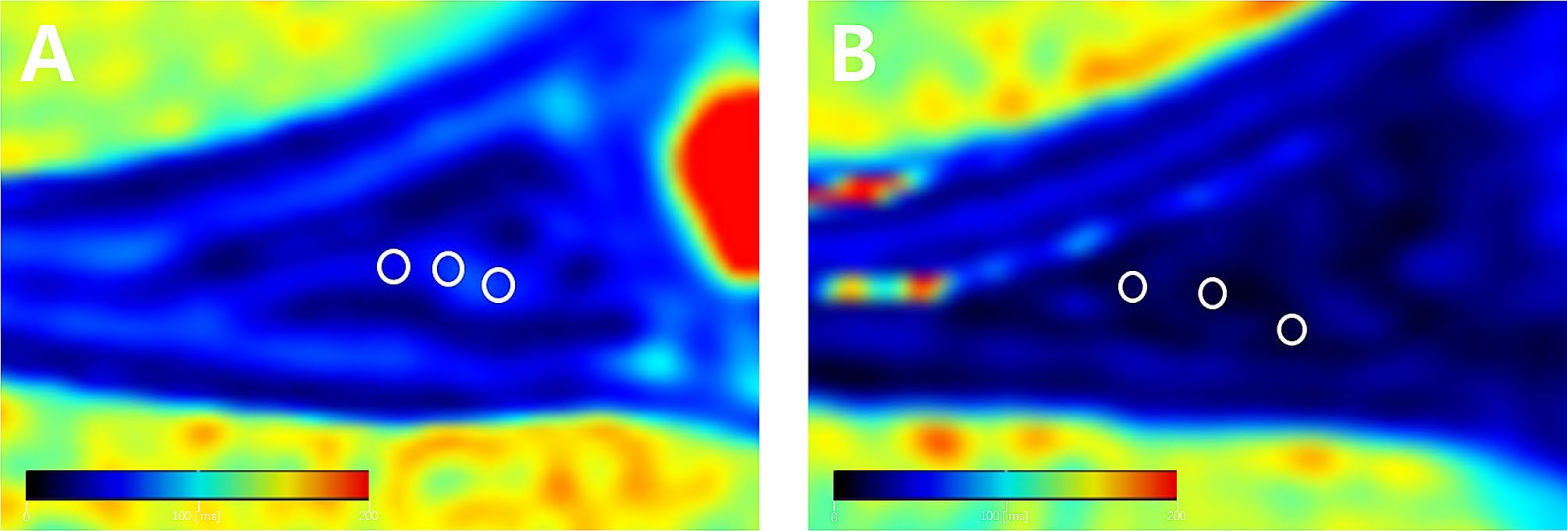
A: Schematic representation of the region of interest of the injury meniscus. B: Schematic representation of the region of interest of the normal meniscus

The SyMRI was post-processed on the main scanning console, and the region of interest was manually drawn on the generated pseudo-color map of quantitative parameters. The T1, T2 and PD values of the abnormal signal region of the meniscus were obtained from the region of interest (ROI) and they were physical properties of tissues and could reflect the situation of different tissues. The ROI was placed at the optimal slice for visualizing the lesion on SyMRI combined with conventional MRI. The principles of ROI delineation of the injury meniscus were as follows: (1) The area of ROI was about 3mm^2^; (2) The ROI should be located in the high signal area of the meniscus on the synthetic T2 mapping, and the interference of the synovial fluid around the meniscus and the transverse knee ligament should be avoided; (3) ROI was fixed at the same injury location of the meniscus (Fig. 2A). The ROI was measured 3 times and the average value was taken. ROI regions of the normal meniscus were randomly selected from the meniscus region of normal subjects for 3 times and the average value was taken (Fig. 2B). In order to ensure the uniformity of measurements, all measurements were performed independently by a radiologist with more than 10 years of experience working in musculoskeletal imaging who was blind to the diagnosis of each participant. All measurements were performed twice, and the time interval between the two measurements was 14 days.

### Statistics

Intraclass correlation coefficient (ICC) was employed to evaluate the consistency of two measurements of each quantitative value. According to the conventional MRI and Stoller grade, all patients were divided into degeneration group and tear group (in the Stoller grade the treatment methods of grade and signals were the same and the signal intensity was similar, so grade and signals were included in the degeneration group), and healthy subjects comprised normal group. One-way ANOVA was used to analyze T1, T2 and PD values between normal group, degeneration group and tear group measured by SyMRI. With arthroscopic findings as the gold standard, One-way ANOVA was utilized to analyze T1, T2 and PD values between 3 groups. McNemar test was conducted to assess statistical significant of differences between the two groups. The receiver operating characteristic curve (ROC curve) was established and the area under the curve (AUC) was employed for assessment. Youden index was employed to determine the optimal threshold value for the diagnosis of meniscal degeneration and tear. All statistical analyses were performed using SPSS v. 13.0 software (Chicago, IL), and *P*=0.05 was considered significant.

## Results

### Subjects

60 (*n* = 60) patients with meniscus injury and 20 (*n* = 20) health subjects were collected in this research. Patients with meniscal injuries were 48 males and 12 females, with a mean age of 32.27 years (range, 18–48 years). The healthy subjects were 8 males and 12 females, with a mean age of 27.6 years (range, 18–30 years). Arthroscopic examinations of 60 patients disclosed 11 cases of meniscal degeneration and 49 cases of meniscal tears. Among the 60 menisci studied, meniscal lesion occurred in 22 cases at the posterior horn of the medial meniscus, 30 cases at the posterior horn of the lateral meniscus, and 8 cases at the anterior horn of the lateral meniscus; no lesion was observed at the anterior horn of the medial meniscus. In cases of meniscal degeneration under knee arthroscopy, the presentation typically involved a seemingly normal or slightly reduced volume, with a meniscal surface exhibiting mild roughness or ciliation. Some patients might also exhibit synovial hyperplasia, a slightly blunted free edge, and maintained tension. Compared to meniscal degeneration, meniscal tears characterized by evident cracks, partial defects with roughened surfaces and cilia. Tension was significantly reduced. Regarding tear types among 49 patients who underwent arthroscopic surgery, there were 22 cases of longitudinal tears, 12 cases of horizontal tears, 5 cases of complex tears, 5 cases of bucket-handle tears,3 cases of root tears and 2 cases of flap tears.

### Consistency study of SyMRI combined with PDWI-FS on meniscus injury grade

Twenty knees were randomly selected for ROI delineation and the time interval between the two measurements was 14 days. The T1, T2 and PD values of the two groups were obtained. The ICC values of T1, T2 and PD values were 0.915 (95%CI: 0.789–0.967), 0.927 (95%CI: 0.815–0.972) and 0.906 (95%CI: 0.766–0.964), respectively.

**Fig. 3:**
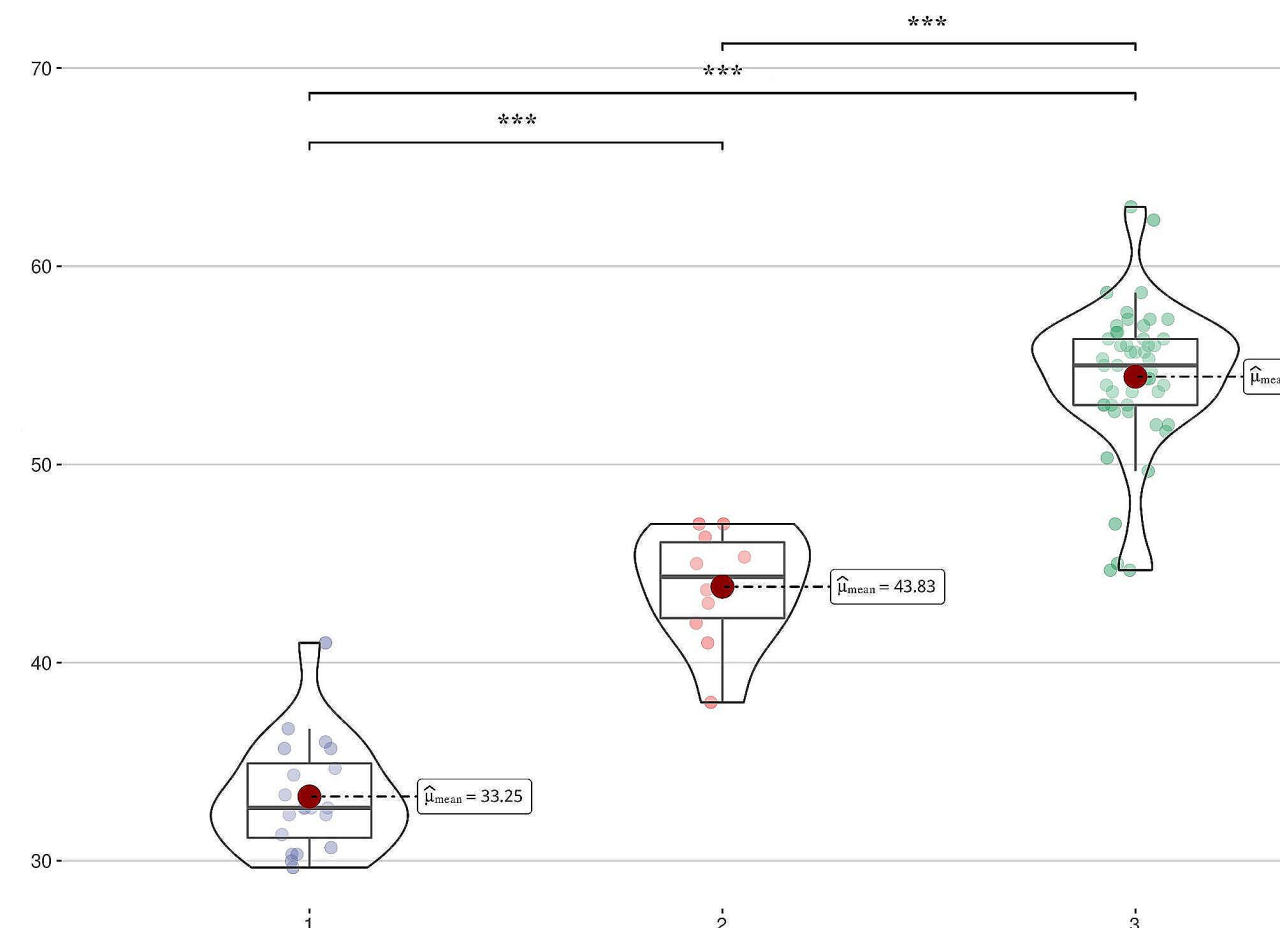
The violin diagram displayed the data distribution of T2 values among the normal group, degeneration group and torn group (***: *P*<0.001)

According to the results of MRI and Stoller grade, there were 20 normal meniscus, 10 degenerated meniscus and 50 torn menisci. Pseudo-color images were automatically generated by post-processing software on the main MR scanning console. There was no statistical difference of T1 or PD value measured by SyMRI among the normal group, degeneration group and torn group. In contrast to T1 and PD value, T2 value was statistically significant among 3 groups (Table 1). Furthermore, when pairwise comparisons were made among 3 groups, T2 values differed significantly, and the difference was statistically significant (*P*=0.001) (Table 1; Fig. 3).

**Table 1:** T1 value, T2 value and PD value in each group and pairwise comparison of T2 value among the 3 groups

Parameters	Normal group	Degeneration group	Torn group	F	P
T1 value (ms)	905.59±282.54	989.63±274.03	1019.45±287.01	2.573	0.08
T2 value (ms)	32.81±2.51	43.83±2.91	54.43±3.69	796.146	<0.01
	32.81±2.51	43.83±2.91	——	——	<0.001
	——	43.83±2.91	54.43±3.69	——	<0.001
	32.81±2.51	——	54.43±3.69	——	<0.001
PD value (pu)	36.04±6.99	37.77±8.56	33.47±9.64	2.057	0.132

### Accuracy study on quantitative analysis of meniscus injury by SyMRI

Using arthroscopic findings as the gold standard, there was no statistical difference of T1 value or PD value measured by SyMRI. However, the difference of T2 value was statistically significant (Table 2). Pairwise comparison revealed that there were statistically significant differences in meniscal T2 values among the 3 groups (Table 2).

**Table 2:** T1 value, T2 value and PD value in each group and pairwise comparison of T2 value among the 3 groups

Parameters	Normal group	Degeneration group	Torn group	F	P
T1 value (ms)	905.59±282.54	1028.21±286.25	1031.81±286.27	0.852	0.431
T2 value (ms)	32.81±2.51	44.85±3.98	54.42±3.82	247.87	<0.01
	32.81±2.51	44.85±3.98	——	——	<0.001
	——	44.85±3.98	54.42±3.82	——	<0.001
	32.81±2.51	——	54.42±3.82	——	<0.001
PD value (pu)	36.04±6.99	33.98±8.50	34.23±9.84	0.219	0.804

The arthroscopic findings showed that 49 cases were meniscal tears and 11 cases were meniscal degeneration. In the diagnosis of meniscal tear by conventional MRI, 4 cases were misdiagnosed and 8 cases were missed (Table 3). In the diagnosis of meniscal tear by SyMRI, 3 cases were misdiagnosed and 2 cases were missed (Table 4).

**Table 3:** Detection of meniscus tear by MRI [n (%)]

MRI detection	Arthroscope detection		Total
	Positive	Negative	
Positive	41 (83.67)	4 (36.36)	45 (75.00)
Negative	8 (16.33)	7 (63.64)	15 (25.00)
Total	49 (81.67)	11 (18.33)	60

MRI, magnetic resonance imaging

**Table 4:** Detection of meniscus tear by MRI [n (%)]

SyMRI detection	Arthroscope detection		Total
	Positive	Negative	
Positive	47 (95.92)	3 (27.27)	50 (83.33)
Negative	2 (4.08)	8 (72.73)	10 (16.67)
Total	49 (81.67)	11 (18.33)	60

SyMRI, synthetic magnetic resonance imaging

**Fig. 4:**
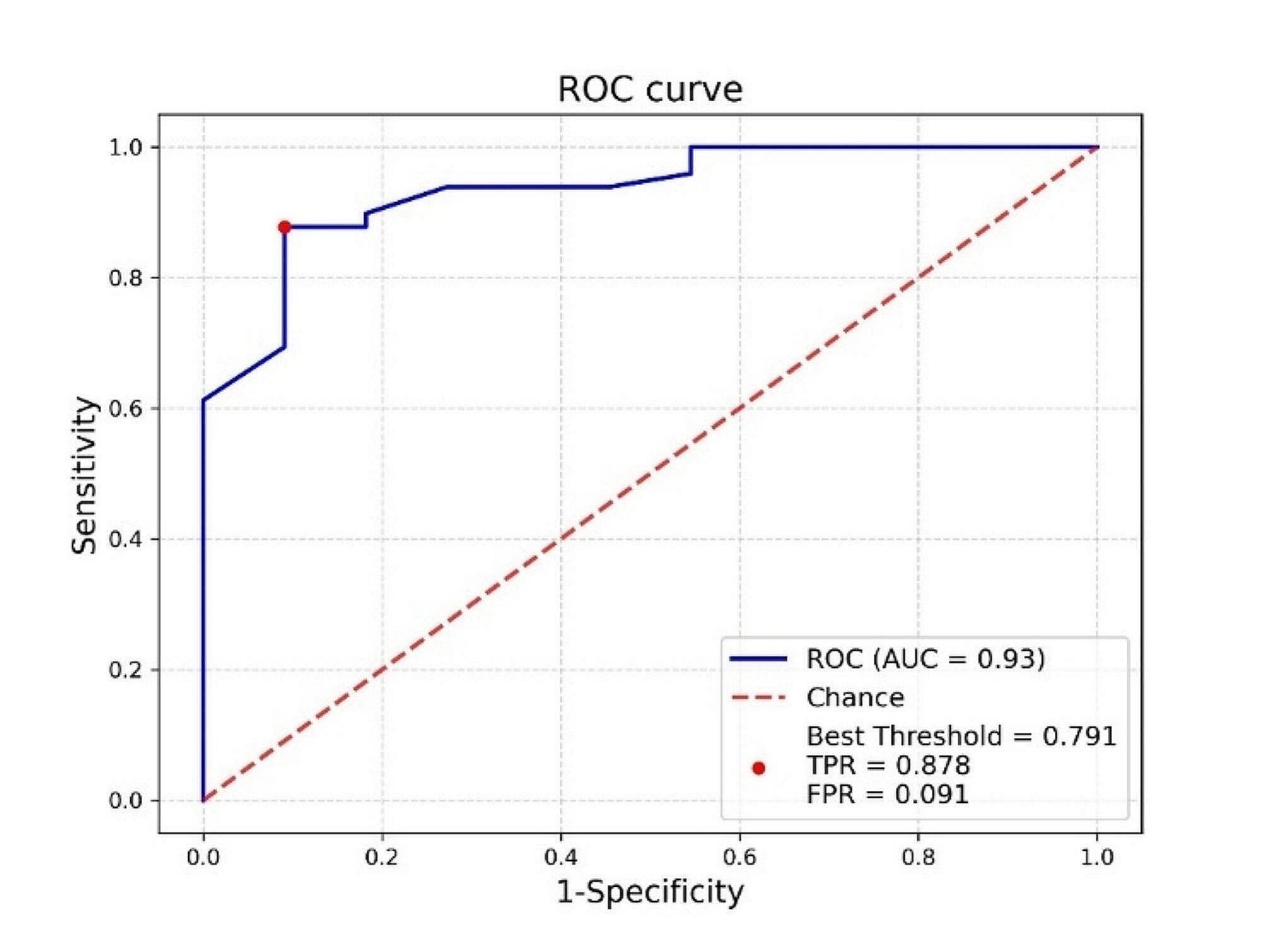
ROC curve of SyMRI in the diagnosis of meniscal tear

McNemar test indicated that there was no significant difference between the results of conventional MRI and arthroscopy, as well as between the results of SyMRI and arthroscopy (*P* > 0.05). The specificity, sensitivity, false negative rate and false positive rate of conventional MRI in the diagnosis of meniscus tear were 63.6%, 83.7%, 16.3% and 36.4%. The specificity, sensitivity, false negative rate and false positive rate of SyMRI in the diagnosis of meniscus tear were 72.7%, 95.9%, 4.1% and 27.3%. The ROC curve revealed that at a T2 value threshold of 51.67 (ms), the maximum Youden index was 0.787 (*P*=0.001), with a sensitivity of 0.878, false positive rare of 0.091, and an AUC of 0.934 (95% Cl: 0.864=∼=1) (Fig. 4).

### Qualitative imaging analysis

**Fig. 5:**
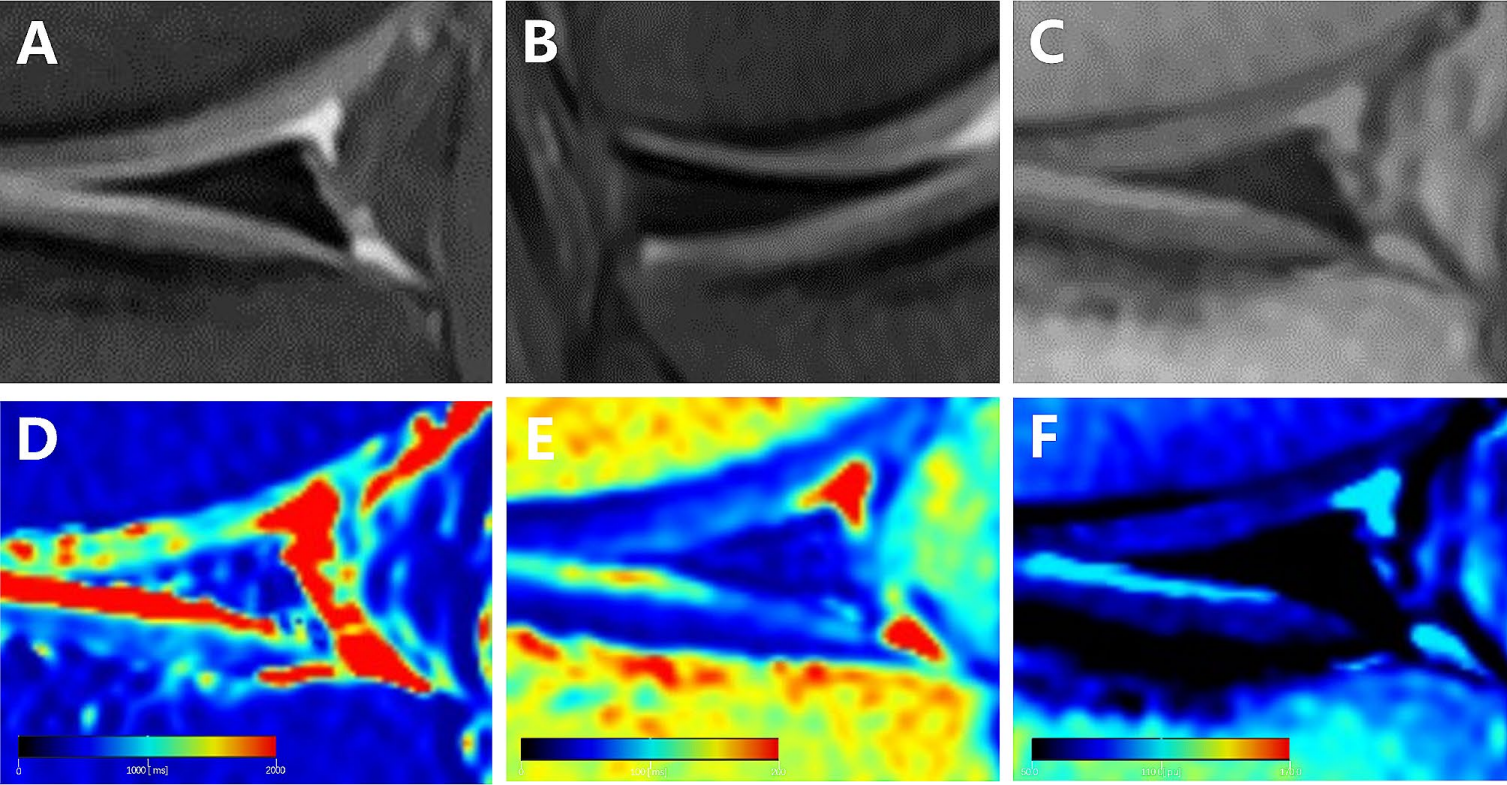
A 30-year-old male subject with posterior horn of the medial meniscus of left knee joint. **A**: Sagittal PDWI; **B**: Coronal PDWI; **C**: synthetic sagittal PDWI; **D**: synthetic sagittal T1 mapping; **E**: synthetic sagittal T2 mapping; **F**: synthetic sagittal PD mapping. There is no abnormal signal conventional MRI (A and B) and SyMRI (C and F) in the posterior horn of the lateral meniscus, but there is abnormal signal on SyMRI (blue color in the meniscus, D and E) within the meniscus

**Fig. 6:**
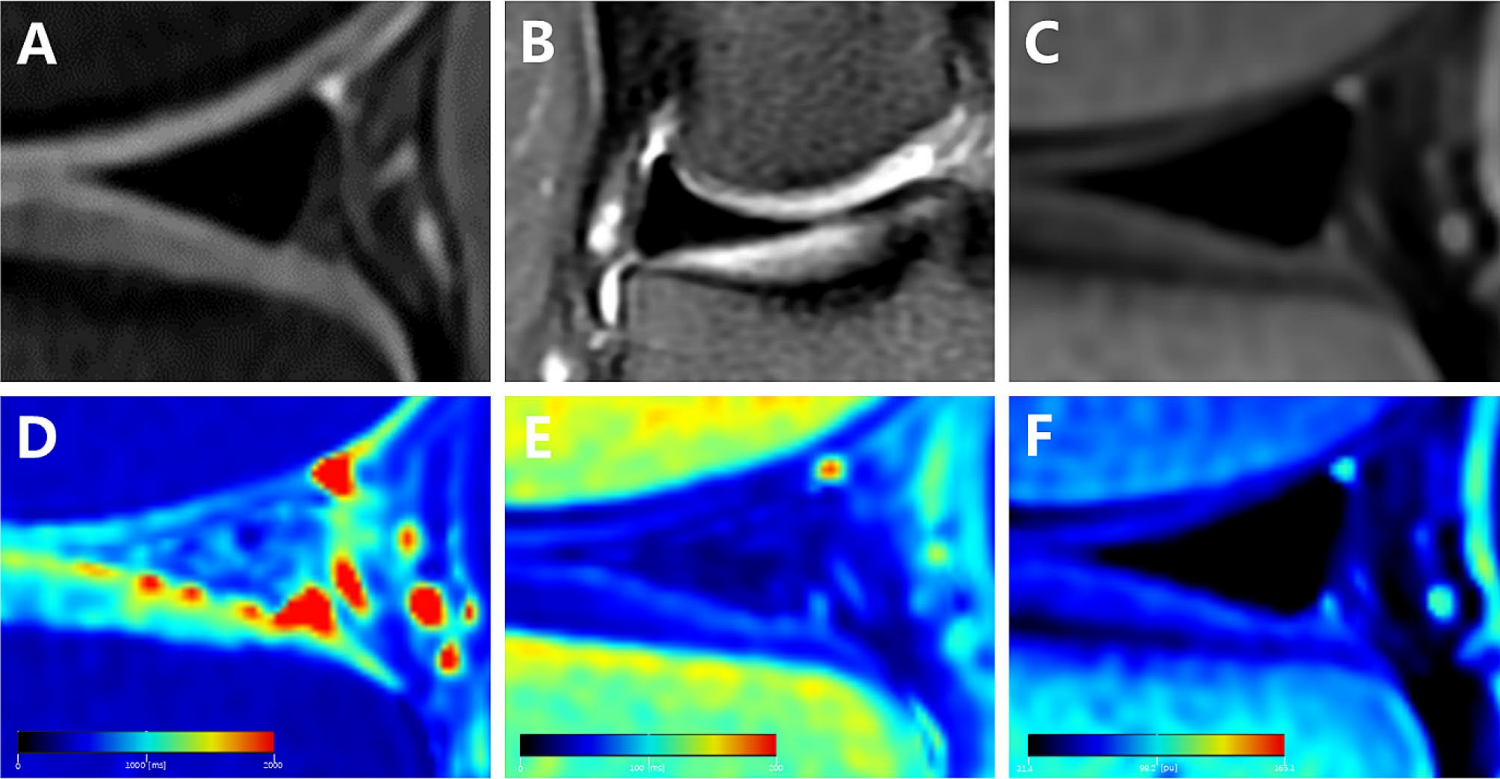
A 24-year-old healthy right knee joint of female subject. **A**: Sagittal PDWI; **B**: Coronal PDWI; **C**: synthetic sagittal PDWI; **D**: synthetic sagittal T1 mapping; **E**: synthetic sagittal T2 mapping; **F**: synthetic sagittal PD mapping. There is no abnormal signal within the posterior horn of the lateral meniscus

Through a comparison of the generated pseudo-color images with PDWI, we observed that the synthetic T1 mapping and synthetic T2 mapping exhibited blue-green or red color levels, signifying meniscus degeneration or tear. However, synthetic PD mapping appeared predominantly blue-green, with minimal variation in color level, making it challenging to identify lesions (Fig. 5). In contrast, the synthetic T1 mapping and synthetic T2 mapping of normal meniscus displayed a relatively uniform dark blue color (Fig. 6).

## Discussion

### Consistency study between stoller grades of meniscus and SyMRI

As a non-invasive imaging technique, MRI plays an crucial part in diagnosing joint diseases. Although conventional MRI can identify meniscus injury based on the signal and shape characteristics, it falls short in assessing compositional changes through histology and detecting early meniscus damage. Currently, extensive research has been conducted on the T2 mapping and T1ρ in joint soft tissue. T2 value and T1ρ value are indices reflecting cartilage tissue components [23]. T1ρ value is particularly sensitive to proteoglycan in cartilage [24]. However, given that the meniscus is primarily composed of collagen fibers with minimal proteoglycan content as fibrocartilage, T1ρ value lacks sensitivity in detecting meniscus lesions.

In this study, SyMRI combined with PDWI-FS was used to conduct a consistency study on the Stoller grade. The higher T2 value was, the higher Stoller grade was, which was also consistent with the results reported in other articles [25]. T2 values provide a quantitatively assessment of collagen concentration and water content in the cartilage matrix [26]. In degenerated menisci, the decrease in collagen fiber content in the extracellular matrix, along with increased joint fluid mobility, leads to prolonged T2 relaxation time. Additionally, torn menisci exhibit the loss of normal shape and increased fluid accumulation in the joint space, extending into the meniscus space [27]. Consequently, the T2 signal intensity in the meniscus tear group was higher than that in the meniscus degeneration group. Although the T2 value of meniscus increases with the Stoller grade, any other relationship with Stoller grades remain inconclusive.

### Study on diagnostic accuracy of meniscus injury by SyMRI

While conventional MRI exhibits relatively high accuracy in diagnosing meniscus injuries, arthroscopy remains the gold standard due to its ability to directly observe morphological changes in the meniscus [28]. T2 values vary during different stages of the meniscus injury process. In the acute phase, impacted or traumatically affected menisci may show higher T2 values. However, as the disease progresses, absorbed water molecules, repaired collagen fibers and scar tissue within the meniscus fissure may lead to a decrease in T2 values [29]. The study also found that there were 3 patients with this misdiagnosis, and the reasons may be as follows: (1) mucoid degeneration of meniscus may lead to more water content of meniscus than that of ordinary degeneration, resulting in an increase of T2 value; (2) The meniscal ligaments, such as the transverse ligament of the knee and the laminofemoral ligament, may be considered as torn meniscus. If the moisture content of the ligament increases due to ligament contusion or tear, the measurement of T2 value may be similar to the T2 value of the meniscal tear; (3) Partial volume effect or artifact of MRI [30]; (4) Meniscus contusion in the acute stage of injury or due to trauma causes will cause T2 values to be higher than meniscal degeneration. In addition, there were 2 patients with missed diagnosis. Probably due to the longer course of the disease and scar formation in the torn meniscus area, the T2 values of SyMRI were not high.

Statistically significant differences of T2 values among 3 groups highlighted the utility of T2 values for quantitative analysis of meniscus injuries. The sensitivity of SyMRI was higher than conventional MRI (95.9% vs. 83.7%), which indicated that SyMRI was more accurate in diagnosing meniscal tear. While the T2 value at the meniscal injury site exceeds 51.67 (ms), the ROC curve analysis yielded an AUC of 0.934, affirming the high diagnostic value of T2 values of SyMRI in meniscus injuries. In clinical practice, SyMRI can be performed on the basis of conventional MRI, which can have high accuracy in the diagnosis of meniscus injury. With the further development of SyMRI research, SyMRI may be gradually applied to clinical practice.

This study presents several limitations. Firstly, the majority of the patients had ACL tear combined with meniscus injury, with a limited representation of other diseases, such as flap or shear lesions and root lesion. Flap tears or root tears was relatively difficult for diagnoses on MRI and the error measurements of SyMRI may occur. Secondly, the meniscal conditions in elderly individuals were more intricate than those in younger counterparts. Additionally, SyMRI itself has constraints: the single scan might result in motion artifacts, particularly in elderly or intolerant patients. The signal-to-noise ratio of synthetic STIR images by SyMRI during MR scan is low, and artifacts from blood vessel pulsation may impact observation.

## Conclusion

T2 value of SyMRI could reflect the Stoller grade, demonstrating that SyMRI had good consistency with conventional MRI. Simultaneously, T2 value of SyMRI could quantitatively analyze meniscus injury. The sensitivity and specificity of SyMRI in diagnosing meniscal degeneration and tear surpassed those of conventional MRI, signifying that SyMRI exhibited superior diagnostic efficiency for meniscal injuries compared to conventional MRI. Moreover, the notable consistency observed between SyMRI and arthroscopy suggests a potential role for SyMRI in guiding clinical diagnoses.

## Data Availability

The datasets used and /or analyzed during the current study are available from the corresponding author on reasonable request with the permission of the Institutional Review Board.

## Abbreviations

SyMRI: synthetic magnetic resonance imaging
MRI: magnetic resonance imaging
MR: magnetic resonance
ACL: anterior cruciate ligament
MDME: multiple dynamic multiple echo
PDWI: proton density-weighted image
UTE: ultrashort echo time
dGEMRIC: delayed gadolinium-enhanced MRI of cartilage
TR: repetition time
TE: echo times
TD: delay times
STIR: short-tau inversion recovery
FLAIR: fluid attenuation inversion recovery
FOV: field of view
ROI: region of interest
ANOVA: analysis of variance
PDWI-FS: fat-saturated proton density-weighted image
T2WI-FS: fat-saturated T2-weighted image
ICC: intraclass correlation coefficient
